# Dementia ascertainment using existing data in UK longitudinal and cohort studies: a systematic review of methodology

**DOI:** 10.1186/s12888-017-1401-4

**Published:** 2017-07-03

**Authors:** Ruth A. Sibbett, Tom C. Russ, Ian J. Deary, John M. Starr

**Affiliations:** 1 Alzheimer Scotland Dementia Research Centre, The University of Edinburgh, 7 George Square, Edinburgh, EH8 9JZ UK; 2 Centre for Cognitive Ageing and Cognitive Epidemiology, The University of Edinburgh, Edinburgh, UK; 3 Department of Psychology, The University of Edinburgh, Edinburgh, UK; 4 Centre for Dementia Prevention, The University of Edinburgh, Edinburgh, UK; 5 Division of Psychiatry, The University of Edinburgh, Edinburgh, UK

**Keywords:** Dementia, Research design and methodology

## Abstract

**Background:**

Studies investigating the risk factors for or causation of dementia must consider subjects prior to disease onset. To overcome the limitations of prospective studies and self-reported recall of information, the use of existing data is key. This review provides a narrative account of dementia ascertainment methods using sources of existing data.

**Methods:**

The literature search was performed using: MEDLINE, EMBASE, PsychInfo and Web of Science. Included articles reported a UK-based study of dementia in which cases were ascertained using existing data. Existing data included that which was routinely collected and that which was collected for previous research. After removing duplicates, abstracts were screened and the remaining articles were included for full-text review. A quality tool was used to evaluate the description of the ascertainment methodology.

**Results:**

Of the 3545 abstracts screened, 360 articles were selected for full-text review. 47 articles were included for final consideration. Data sources for ascertainment included: death records, national datasets, research databases and hospital records among others. 36 articles used existing data alone for ascertainment, of which 27 used only a single data source. The most frequently used source was a research database. Quality scores ranged from 7/16 to 16/16. Quality scores were better for articles with dementia ascertainment as an outcome. Some papers performed validation studies of dementia ascertainment and most indicated that observed rates of dementia were lower than expected.

**Conclusions:**

We identified a lack of consistency in dementia ascertainment methodology using existing data. With no data source identified as a “gold-standard”, we suggest the use of multiple sources. Where possible, studies should access records with evidence to confirm the diagnosis. Studies should also calculate the dementia ascertainment rate for the population being studied to enable a comparison with an expected rate.

**Electronic supplementary material:**

The online version of this article (doi:10.1186/s12888-017-1401-4) contains supplementary material, which is available to authorized users.

## Background

As the global population ages and dementia rates increase, further research is required in order to reduce the impact on the individual and on society [1, 2] Key aspects of current dementia research include causation, risk factors, early detection, and prevention. In order to investigate such factors robustly – and avoid reverse causality – studies need to consider subjects prior to disease onset. Whether such studies concentrate on the entire life course or on a limited period prior to dementia onset, completing data collection prospectively can be time-consuming and costly. Recruiting those who already have a diagnosis in order to consider life-course risk and protective factors is limited by the potential inaccuracy or incompleteness of information recalled by participants and carers.

In order to overcome such limitations, the use of previously collected data is key. The value of existing data sets is demonstrated by the launch of The Farr Institute of Health Informatics Research in the UK, aimed at optimising the use of health records in research by facilitating the safe and secure use, and linkage of, electronic patient records, research data and routinely collected data [3].

Using existing data in dementia research is not unusual. Death certificates are often utilised to complement clinical follow-up methods where study participants are lost to follow up [4, 5]. Although studies have used existing data for the purposes of dementia ascertainment, to our knowledge no review has been produced in order to collate and consider the various methods described. As a result, there is no clear guidance or standard to follow when designing a study using existing data for dementia ascertainment. The aim of this systematic review is therefore to provide a narrative account of the dementia ascertainment methods using existing data sources described in the literature, in order to provide evidence for potential approaches in future research. It should be noted that this review focuses on ascertainment from sources of existing data, rather than on the specific dementia criteria utilised by each study. This review is specifically aimed at providing a basis for dementia ascertainment methods for studies based in the UK, where there are highly developed systems allowing the capture of health outcomes from a variety of sources. This review will therefore not consider studies based out-with the UK, as datasets vary widely between countries and health systems. It is however likely that some of the data sources considered in this review will have an equivalent in other countries and so our conclusions will have relevance outside the UK.

## Methods

The Preferred Reporting of Items for Systematic Reviews and Meta-Analyses (PRISMA) statement was used to guide the conduct and reporting of the present systematic review [6].

### Selection criteria

The ‘PICOS’ approach (population, intervention, comparator, outcome, study design) was adopted in order to define the study question and build an appropriate search strategy. Given that this review would focus on methodology, the intervention and comparator were not applicable. The population (P) would be a UK-based cohort or population group, the outcome (O) would be the dementia ascertainment method and sources of existing data, and the study design (S) would be observational. This review aimed to guide future studies performing dementia ascertainment using existing data within the UK. This review will not consider populations out-with the UK as health data and systems vary from that which is available in the UK.

### Data sources

Scholarly articles for inclusion in the review were identified through searching four separate electronic databases determined to be appropriate for dementia ascertainment methodology. The following databases were included: MEDLINE (from 1946), EMBASE (from 1980), PsychINFO (from 1987) and Web of Science (from 1900). The literature search took place on the 28th December 2015.

### Search strategy

The study authors (who have expertise in dementia) developed the search strategy with input from a research librarian experienced in systematic review methodology. Each search included terms relating to: a) dementia; b) the UK; and c) longitudinal or cohort study type. The full electronic search strategy for MEDLINE is detailed in Additional file 1. No limitation parameters were used. The inclusion and exclusion criteria for this review aimed to strictly limit included papers to those using previously collected data for dementia ascertainment. Specifically, this required the exclusion of any paper where dementia status was used to select participants or where dementia was determined by prospective clinical review. The exclusion criteria needed to be extensive given the broad terms used for the initial literature search. Such broad search terms were required given that the area of interest was methodology, rather than the primary topic of a study.

The inclusion criteria for review articles were: a) Longitudinal or cohort studies applying retrospective dementia ascertainment methods; b) Dementia cases must be ascertained from a defined larger cohort/population; c) Ascertainment may be an outcome, or ascertainment may be performed in order to determine a cohort of participants with dementia; d) Dementia diagnoses were ascertained using existing data (in part or in full). Existing data included that which was collected routinely and that which was collected for previous research.

The exclusion criteria were: a) Study population based outside the United Kingdom; b) Articles published in non-English language; c) Participant self-referral/other referral to studies following advertising for persons with dementia; d) Participant/carer/health service response to census/survey; e) Participants included based on known neuropathological diagnosis of dementia; f) Direct referral of participants from NHS/voluntary services following advertising/request for referral of persons with dementia; g) Study participants recruited from hospital wards, outpatient clinics (or referrals to the same) or other services, unless documented that records/other existing data used to select cases; h) Study participants selected from an existing register of dementia cases, study/research centre, memory or old age psychiatry clinic patients, people prescribed cholinesterase inhibitors or dementia carers; i) Studies where dementia was not the primary condition or disease of interest, or at least of equal weight to another condition; j) Animal models of dementia; k) Simulated cohorts; l) Ascertainment not for dementia diagnosis (i.e. cognitive decline ‘suggestive of dementia’, cognitive impairment); m) Systematic reviews (any systematic review on this specific topic would be included, but reviews producing summary data from several studies without any primary description of dementia case ascertainment would be excluded) /meta-analyses/case reports – i.e. any non-longitudinal or cohort study; n) Studies where dementia cases were ascertained entirely at baseline and/or prospectively in a clinical assessment setting; o) posters or abstracts; p) unclear description, additional duplicates or errors in citation.

### Study selection

References were exported to and managed using the reference management software package Endnote X7.5. The results from each database search were compiled and any duplicates removed. Duplicates were identified and removed by the ‘find duplicates’ function within the Endnote software. Additional duplicates were then identified and removed manually. Records returned by the literature search were excluded sequentially. In an initial phase of screening, titles and abstracts were reviewed for eligibility by the first author, according to the inclusion and exclusion criteria. The threshold for inclusion at this phase was purposefully low, to prevent the exclusion of any relevant study. The full-texts of articles remaining following the initial screening were obtained and independently scrutinised by the first and second authors, according to the same inclusion and exclusion criteria. Any discrepancies between the final full-text lists for inclusion between the first and second authors were discussed and agreed upon at a meeting. It was planned that any disagreements persisting following the meeting would be discussed with a third author.

Where the list of eligible full-text articles included more than one article by the same study group or author(s), and described the same ascertainment methodology, we included only the article with the most comprehensive description of the methodology, and excluded all the others. This was done in order to prevent bias in our findings. We aimed to prevent double-counting of a single study which had given rise to multiple research outputs – where a single group or author had published multiple articles using the same methodology from a single project. Including all such articles would risk making it appear that a specific methodology or data source was used more frequently and more widely than in reality.

### Data collection

The data extracted from the eligible full-text articles included: the author(s), the journal reference (including year of publication), the study topic or aim, whether dementia ascertainment was the outcome or where it was ascertained to form a cohort for further study, the source(s) of existing data for dementia ascertainment, the criteria for dementia ascertainment, and whether there was any validation procedure or comparison with expected dementia rates. Data were also extracted for the purpose of evaluating the quality of the methodology description, as detailed below.

### Quality measure

A quality tool was developed by the authors in order to evaluate the description of dementia ascertainment methodology within each included article. The components of the quality measure were based on whether the paper contained sufficient information such that an incidence or prevalence rate could be calculated and the ascertainment rate could be compared with another population. The components of the quality measure considered the description of: a) the size of the baseline population; b) the age of the baseline population; c) the sex of the baseline population; d) the source of the baseline population; e) the ascertainment procedure and f) the date or time period studied. Each article was given a quality score, with a higher score indicating a higher-quality description of dementia ascertainment methodology. The maximum score was 16 and the full details of the quality tool are shown in Table 1.

**Table 1:** Quality Tool

	Quality tool components
1	Baseline population size A) Exact number (score = 3) B) Approximate number (score = 2) C) Other description of size (score = 1) D) Not specified (score = 0)
2	Baseline population age AI) Exact age range specified for total population (score = 4) AII) Broad age range specified for total population (score = 3) BI) Exact age range specified for analyses (score = 2) BII) Broad age range specified for analyses (score = 1) C) Not specified (score = 0)
3	Baseline population sex A) Specified (score = 1) B) Not specified (score = 0)
4	Baseline population A) Named with description (score = 2) B) Named only (score = 1) C) Not specified (score = 0)
5	Dementia ascertainment AI) Sources and specific criteria clearly described (score = 3) AII) Sources and specific criteria less clearly described (score = 2) B) Sources named but no specific criteria described (score = 1) C) Unclear/ not described (score = 0)
6	Dementia cases A) Number plus comparison to expected/ documented rate (*external UK comparison*) (score = 2) B) Number only (score = 1) C) Not specified (score = 0)
7	Time/ period/ date A) Specified B) Not specified/ unclear

Where more than one eligible article reported results based on the same study population and the same ascertainment method, the article with the highest score for the quality of description of dementia ascertainment methodology would be included and the others excluded from the final review. These exclusions would be important in order that studies or research groups with a high output of articles from the same study, using the same ascertainment methodology, did not bias the review findings. Specifically, we wanted to avoid certain methods appearing to be frequently used within the UK when the same study group was actually using them multiple times.

## Results

### Article selection

**Fig. 1:**
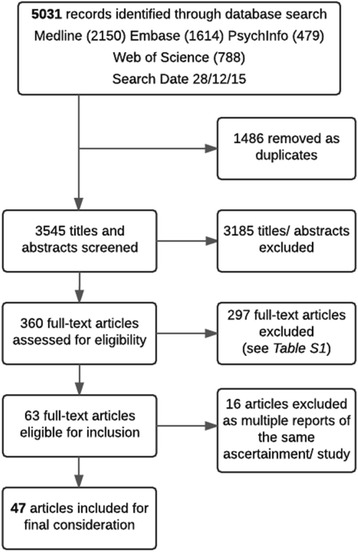
Study Flow Design

A total of 5031 citations were identified by the four separate database searches, including 2150 from MEDLINE, 1614 from EMBASE, 479 from PsychInfo and 788 from Web of Science. After collating the search results, 1486 duplicates were removed. The title and abstract for each of the remaining 3545 records was screened for suitability and it was determined that 360 full-text articles required full-text review. Of those selected for full-text review, it was agreed that 63 articles met the criteria for inclusion. A flow chart of study selection is shown in Fig. 1.

The reasons for the exclusion of papers following full-text review differed between the first and second authors, largely since several papers could have been excluded for multiple reasons. Rather than excluding articles based on multiple exclusion criteria the authors made exclusions based on a primary exclusion criteria. Despite the differences, both authors excluded the same papers. The reasons for exclusion are therefore not detailed in Fig. 1, but presented in Additional file 2: Table S1. The most frequent reasons for the exclusion of full-text articles (combined total number excluded by both authors) were: cases ascertained entirely through baseline or prospective clinical assessment or new data only (*n* = 103), not a longitudinal or cohort study (*n* = 125) and participants recruited directly from hospitals, clinics or other service (*n* = 102). It would be expected that a large number of ineligible or irrelevant articles were returned by the literature search owing to the broad search parameters used in order to identify a methodology rather than a specific study topic. Many such articles also passed through the first phase of screening because the title and abstract did not contain sufficient detail regarding the study methodology in order for eligibility to be determined.

A number of the eligible articles reported results based on the same study population and the same ascertainment method was described multiple times. We identified nine groups of articles reporting the same methodology and *n* = 16 articles were excluded on this basis. Details of the excluded articles are shown alongside the articles chosen for inclusion in Additional file 3: Table S2.

Following this process, 47 papers remained for consideration in this review. An overview of the characteristics of the included studies can be seen in Table 2. The table includes: the topic of the article; whether dementia ascertainment was an outcome of the study or whether ascertainment was performed to build a cohort of subjects with dementia upon which further study was carried out; whether existing data was used in full or in part; the existing data sources utilised; the diagnostic criteria used; whether the article included a validation study of dementia ascertainment or a comparison between observed rates and expected rates; and the total score achieved when the quality tool was applied to each article. We determined that existing data was used in part when new data was used in any way to support ascertainment. New data included clinical assessment, informant interview and contact with services for information.

**Table 2:** Articles included in final review

Author	Study topic	Dementia outcome/ cohort^a^	Existing data use	Data sources	Dementia criteria	Validation/ comparison^b^	Quality score
Baker et al. [56]	Hip fracture risk and mortality in AD	Cohort	Full	THIN^e^	Diagnostic code for AD, OR Prescription for an AD defining drug.	No	9
Brayne et al. [31]	Dementia at death and prevention	Outcome	Part	Death certificates	ICD-10 codes for dementia.	Yes	15
Brayne et al. [30]	Neuropathological correlates of dementia	Outcome	Part	Death certificates	DSM-IV criteria using data from all sources including death certificates	Yes	15
Chen et al. [32]	Health care resource utilisation in AD	Cohort	Full	CPRD^f^	Diagnosis of AD.	No	8
Clarke et al. [4]	Dementia incidence	Outcome	Part	Death certificates, hospital case notes	‘Diagnostic information’ from both sources.	Yes	14
Cook et al. [37]	Incidence of stroke and seizure in AD	Cohort	Full	THIN^e^	Read codes for AD.	No	9
Crugel et al. [7]	Antipsychotic use in dementia	Cohort	Part	Electronic clinical records for trust (Rio)	Confirmed ICD-10 diagnosis of dementia.	No	7
Doll et al. [42]	Smoking and dementia	Outcome	Full	Death certificates	Dementia ‘mentioned’ on death certificate.	Yes	11
Dregan et al. [57]	Inflammation, related therapy and dementia	Outcome	Full	CPRD^f^	Diagnostic codes for dementia.	No	13
Goh et al. [13]	Angiotensin receptor blockers and dementia	Outcome	Full	CPRD^f^	Read codes for dementia.	No	14
Grant et al. [58]	Incontinence and dementia	Cohort	Full	THIN^e^	One or more codes dementia codes, OR Two or more prescriptions for drugs to treat dementia.	No	14
Guthrie et al. [16]	Psychotropic drug prescribing in dementia	Cohort	Full	SPICE-PC^h^	Read codes for dementia, OR Prescription of acetylcholinesterase inhibitor.	Yes	14
Heath et al. [9]	Vascular comorbidities in early onset dementia	Outcome	Full	General practice research dataset (unnamed)	Read codes for dementia, OR Prescription of acetylcholinesterase inhibitor.	Yes	16
Houttekier et al. [59]	Place of death in dementia	Both	Full	Death certificates	ICD-10 codes for dementia.	No	13
Imfeld et al. [11]	Epidemiology, comorbidities and medication in AD & VD	Outcome	Full	GPRD^g^	Stage I: read code for dementia, OR, prescription for acetylcholinesterase inhibitor. Stage II: algorithm for AD or VD (based on DSM-IV, NINCDS-ADRDA, NINDS-AIREN, NICE & SIGN).	Yes	9
Karlinsky et al. [60]	AD in twins	Outcome	Full	Hospital twin register, hospital case notes	Stage I: discharge diagnosis suggestive of dementia on register, Stage II: case note diagnosis of AD according to DSM-III-R criteria.	No	10
Keenan et al. [29]	Glaucoma and AD/ VD	Both	Full	Hospital Episode Statistics	ICD-10 diagnostic code for AD or VD.	No	15
Kehoe et al. [35]	Angiotensin targeting anti-hypertensives, mortality and hospitalisation in dementia	Cohort	Full	GPRD^g^	Read codes for dementia.	No	9
Lu et al. [61]	Gout and risk of AD	Outcome	Full	THIN^e^	Diagnostic codes for AD.	No	14
Martinez et al. [62]	Trends in antipsychotic drug use in dementia	Cohort	Full	CPRD^f^	Read codes for dementia.	No	9
McCarthy et al. [33]	Experience of dying with dementia	Both	Full	Death certificates	Dementia diagnosis coded.	No	8
McGonigal et al. [22]	Epidemiology of pre-senile AD	Outcome	Full	^d^ISD Scotland data for psychiatric hospitals, hospital records	Stage I: diagnostic codes for dementia in SMR, Stage II: NINCDS-ADRDA criteria and Hachinski score applied to records.	Yes	13
Morgan et al. [63]	Physical activity in midlife and dementia	Outcome	Part	Death certificates	Mention of dementia in either part one or part two of death certificate (used in conjunction with new data at consensus)	No	15
Newens et al. [26]	Ascertainment, incidence, prevalence & survival in pre-senile AD	Outcome	Part	Electronic hospital information systems (^c^HAA, MHE, Körner), neuroradiology records, case notes	Stage I: potential cases identified by ICD-9 codes from information systems, referrals for CT with dementing process, (*and contact with services*). Stage II: case notes for all examined for DSM-III-R criteria for dementia, then algorithm for pre-senile AD.	Yes	13
Palmer et al. [8]	Dementia and occupational exposure to solvents	Cohort	Full	CT records, hospital case notes	Exact criteria not specified.	No	7
Pendlebury et al. [5]	Risk of dementia after TIA and stroke	Outcome	Part	Clinical records (hospital & GP), death certificates	Recorded diagnosis of dementia in primary care record, DSM-IV criteria met after searching the primary care record or dementia listed on death certificate.	No	14
Perera et al. [34]	Response to acetylcholinesterase inhibition in dementia	Cohort	Full	Electronic medical records, hospital trust case register	Patients receiving Acetylcholinesterase Inhibitor.	No	8
Qizilbash et al. [14]	BMI and risk of dementia	Outcome	Full	CPRD^f^, death certificates	Dementia term recorded in CPRD*, OR Dementia diagnosis on death certificate.	No	13
Rait et al. [17]	Survival in dementia	Both	Full	THIN^e^	Read codes for dementia.	Yes	10
Renvoize et al. [23]	Prevalence of young onset dementia	Outcome	Part	Local computerised information systems, medical and social care records	Stage I: discharge diagnosis of dementia in computer system, Stage II: “criteria for dementia” from notes (criteria not specified)	Yes	13
Reyniers et al. [18]	International variation in place of death in dementia	Cohort	Full	Death certificates	ICD-10 dementia diagnosis.	No	10
Russ et al. [10]	Geographical variation in dementia (Scottish sub-study only)	Outcome	Full	^d^ISD Scotland data, death certificates, records for a nursing home medical practice	ICD-9 & 10 codes for dementia from ^d^SMR data and death certificates, and dementia status reported by a medical practice.	Yes	16
Ryan [27]	Hospital incidence rates in dementia	Outcome	Full	^d^ISD Scotland data.	ICD-8 & 9 codes for dementia.	Yes	14
Sampson et al. [24]	Differences in care for those with and without dementia	Cohort	Full	Medical notes	Formal diagnosis of dementia recorded.	No	13
Seshadri et al. [12]	Oestrogen replacement therapy and the risk of AD	Both	Full	GPRD*, GP records	Stage I: computer diagnosis of dementia, Stage II: records for each reviewed to confirm diagnosis based on NINCDS-ADRDA criteria.	Yes	14
Shah et al. [25]	Dementia and influenza vaccination uptake	Outcome	Full	THIN^e^	Read codes for dementia.	Yes	12
Sleeman et al. [19]	Place of death and dementia	Cohort	Full	Death certificates	ICD-10 codes for dementia.	No	8
Sorahan et al. [20]	AD, MND and PD in magnetic field exposure	Outcome	Full	Death records	ICD 9 & 10 codes for dementia.	No	12
Stephens et al. [28]	Prescribing of antipsychotics in dementia on the acute general hospital	Cohort	Full	Hospital Episode Statistics, linked to pharmacy records	ICD-10 code diagnosis of dementia.	No	9
Su et al. [64]	MMSE as a predictor of mortality	Cohort	Full	Hospital trust case register	ICD-10 codes for dementia.	No	14
De Vries et al. [65]	Dementia deaths in a hospice	Outcome	Full	Hospice case notes/ referrals	Formal diagnosis of dementia in the notes, other evidence in the notes (no exact criteria given).	Yes	12
Whalley et al. [66]	Effects of anticholinergic drugs on cognition	Outcome	Part	Clinical records, imaging results	Diagnoses based on ICD-10 criteria, using *all source* data.	No	15
Whalley et al. [67]	Childhood mental ability and dementia (Late onset sub-study only)	Outcome	Part	Psychiatric case register, hospital records	Stage I: diagnosis of dementia on register, Stage II: ICD-10 diagnosis of dementia in case notes.	No	14
White et al. [68]	Dementia, walking outdoors and getting lost	Outcome	Full	Missing persons reports	Presence of a key dementia term.	No	11
Wilcock et al. [69]	Educational intervention to improve the management of dementia	Outcome	Full	Electronic GP records	Search for dementia in records- exact criteria not specified.	No	10
Woodburn et al. [70]	Features of early onset dementia in Scotland	Cohort	Part	Psychiatric case register, neurology service records, case notes	Stage I: ICD-9 codes for dementia in register, Stage II: Feighner criteria for Organic Brain Syndrome applied to notes (criteria for neurology records not specified)	No	12
Wotton et al. [71]	Obesity and subsequent dementia	Outcome	Full	Hospital Episode Statistics linked with death records	ICD-9 & 10 codes for dementia.	No	14

^a^: Was dementia ascertainment performed as the study outcome, or was dementia ascertainment performed in order to form a dementia cohort for which another outcome was determined

^b^Did the paper perform a validation study for dementia ascertainment or compare incidence/ prevalence rate to expected rate

^c^ *HAA*: Hospital Activity Analysis; MHE: Mental Health Enquiry system; Körner: Korner Episode Statstics (hospital information systems)

^d^ *ISD* Scotland: Information Services Division Scotland (holds national datasets (SMR: Scottish Morbidity Records) of health outcomes/ diagnoses)

^e^ *THIN*: The Health Improvement Network; ^f^ *CPRD*: Clinical Practice Research Datalink; ^g^ *GPRD*: General Practice Research Database; ^h^ *SPICE-PC*: Scottish Programme for Improving Clinical Effectiveness- Primary Care

### Sources of data

**Fig. 2:**
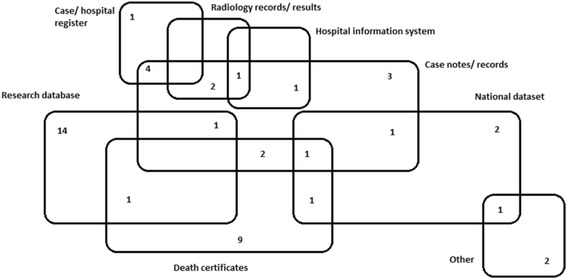
Sources of Existing Data for all Included Articles

The sources of existing data described by the eligible articles were numerous and included general practice research databases (*n* = 16), case notes or records (*n* = 16), death certificates (*n* = 14), case registers (*n* = 5), national datasets (*n* = 6), electronic hospital information systems (*n* = 2), radiology records (*n* = 3), hospice records (*n* = 1), pharmacy records (*n* = 1) and missing person records (*n* = 1). Of the 47 included papers, 31 (66%) used only a single source of existing data for the purpose of ascertainment (Table 2). The highest number of different data sources used was three. The most commonly used data source in studies using a single data source was a research database, such as the General Practice Research Database (GPRD), the Clinical Practice Research Datalink (CPRD), The Health Improvement Network (THIN) or the Scottish Programme for Improving Clinical Effectiveness- Primary Care (SPICE-PC) (*n* = 14). The next-most used data source in articles using a single data source was death certificates (*n* = 9). When considering all included papers, the research database and case records or notes were equal as the most frequently used sources (*n* = 16), but in those papers using multiple sources only, case notes or records were the most frequently utilised source of existing data (*n* = 13). Figure 2 illustrates the source or sources of existing data used by each of the 47 articles.

Of the 36 papers using existing data only for the purpose of dementia ascertainment (i.e. no clinical component), 27 (75%) used only a single data source and the most frequently utilised data source was the research database (*n* = 14). Including the articles that only used existing data and used single or multiple sources, the research database remained the most frequently used (*n* = 16), but considering those using multiple sources separately, case notes were the most frequently used (*n* = 6).

The criteria utilised by the articles to determine a diagnosis of dementia from the data sources was varied. Most studies using death certificates for dementia ascertainment extracted a diagnosis based on the condition having been recorded on any part of the certificate. Others stated that dementia cases were recognised by specific ICD-10 codes listed on the certificate. Evidence from death certificates were also used in combination with evidence from other sources to determine cases. Case records were in many cases reported to be examined by a specialist medical doctor with training in dementia. Researchers often searched the case records for evidence to meet a specific diagnostic criteria, such as DSM-IV, ICD-10 or NINCDS-ADRDA criteria. In other cases, dementia diagnosis was taken as a formal diagnosis or mention of a diagnosis within in the notes. Researchers employed a number of different techniques when using databases to ascertain dementia diagnoses. These included searching the database for diagnostic codes, Read Codes derived from Quality and Outcomes Framework (QOF) codes, recorded diagnoses or prescriptions for dementia defining drugs. Some studies applied diagnostic criteria to evidence within the records. Other studies developed algorithms that combined a number of different criteria. Details of the criteria utilised by each study is shown in Table 2.

### Quality measure

The quality measure was primarily a means of evaluating the description of the ascertainment methodology and the sources of data used. We identified significant discrepancies between the detail provided in the methodology of articles and the quality measure results give an indication of the completeness or lack of information provided by the authors. The quality measure is therefore closely related to our reporting of the sources used and how dementia was ascertained from each.

The breakdown of the quality measure results for each article are shown in Additional file 4 *:* Table S3*.* When an article included sub-studies where dementia was included as an outcome in one study and for a dementia cohort study in another, the quality measure was performed on the outcome study due to the more specific detail included. Where only one sub-study met the inclusion criteria for this review, the results listed considered only the eligible sub-study. Overall, the quality scores ranged from 7/16 [7, 8] to 16/16 [9, 10] and the mean score achieved across all 47 papers was 12.0 (SD: 2.6). Quality scores were lower for studies using existing data alone for ascertainment (*n* = 36; mean = 11.5 (SD 2.6)), compared with studies using existing data in addition to other methodologies (*n* = 11; mean 13.4 (SD 2.3)) (*p* < 0.05). There was also a significant difference (*p* < 0.001) in quality scores between studies where dementia was either an outcome or both an outcome and the basis of forming a cohort (*n* = 31; mean = 13.0 (SD 2.1)), and studies where dementia ascertainment was performed to build a cohort (*n* = 16; mean = 10.2 (SD 2.6)).

The quality measure also included whether a validation study or comparison with expected dementia rate was performed. Given the importance of validating an ascertainment methodology in order to determine its effectiveness we have expanded on this further, as follows.

### Studies reporting a validation procedure

Of the 47 papers included, relatively few performed a validation study for dementia cases or compared ascertainment to previously documented rates. Imfeld et al. [11] completed a validation procedure for the algorithm used to identify cases within the General Practice Research Database (GPRD), and found up to 80% of Alzheimer’s disease cases and up to 75% of vascular dementia cases were confirmed by GP questionnaire responses. Seshadri et al. [12] completed a validation of Alzheimer’s disease cases identified using code algorithms for dementia or Alzheimer’s disease within GPRD and confirmed only 48% cases as either possible or probable Alzheimer’s disease according to NINCDS-ADRDA criteria. Authors did however report a much higher validation rate of 83% for those identified specifically as Alzheimer’s disease cases within the GPRD, and for whom there was adequate data for validation [12]. Imfeld et al. [11] reported that the incidence rates of Alzheimer’s disease found in their study, were three to six times lower than those found in previous European studies. Goh et al. [13] and Qizilbash et al. [14] did not perform any validation study, but referred to the above-mentioned work by Seshadri et al. [12, 15]. Using the Scottish Programme for Improving Clinical Effectiveness- Primary Care (SPICE-PC) database, Guthrie et al. [16] found that the prevalence of dementia was only about half of that found in epidemiological studies. Recording was found to be particularly poor in older age groups. Rait et al. [17] used The Health Improvement Network (THIN) database and on comparison with incidence rates demonstrated by the *EURODEM* study and Cognitive Function and Ageing Study (*CFAS),* the incidence rates found by Rait et al. were shown to be significantly lower than would have been expected [17]. Heath et al. [9], who did not name the research database used, reported the prevalence in their population to be close to the middle of the range of previous estimates.

The under-reporting of dementia on death certificates was noted in articles in this review [10, 18–20]. Doll et al. [21] compared their findings to European statistics (EURODEM) and determined that they had only recorded 30% of dementia cases from death certificates. Russ et al. [10] found that compared to using multiple sources, death certification alone missed approximately 16–18% of dementia cases. The same study found that general practice records did not identify all cases identified by record linkage [10].

McGonigal et al. [22] tested the assumption that most patients with pre-senile Alzheimer’s disease are known to psychiatric services. After consulting further data sources including death certificates, general hospital and neurology service records, and opinions within the medical community, the authors reported that approximately 97% of participants identified as having pre-senile dementia in their study were indeed cared for within psychiatric services [22]. Overall, McGonigal et al. [22] found that the annual incidence rates of pre-senile Alzheimer’s disease, determined using hospital records, within their study population were comparable to annual incidence rates quoted by a national study using different ascertainment methods. It should be noted that some 11% of hospital records requested by McGonigal et al. [22] were either lost or contained insufficient data to apply the diagnostic criteria. If the proportion of probable dementia in missing records was the same as for the available records, 12% of cases would have been missed as a result [22]. Crugel et al. [7] did not perform any validation study but did note that it was known that the number of dementia cases identified using their electronic record system was lower than the number known to the hospital trust. Renvoize et al. [23], who used local computerised medical and social records for case identification, found a prevalence rate consistent with previous studies. Pendlebury et al. [5] state an awareness of under-recording of dementia diagnoses in primary care records and Sampson et al. [24] report that dementia is under-diagnosed in the acute hospital setting. Shah et al. [25] also acknowledge the deficiencies in recognition and recording in primary care, and note that the prevalence found in their study is lower than what might be expected, based on epidemiological surveys. Newens et al. [26] used hospital information systems and clinical records to ascertain the incidence and prevalence of early onset dementia. The authors reported a similar prevalence rate to rates documented elsewhere.

Ryan [27] acknowledged potential diagnostic and clerical errors within the Scottish Morbidity Records (SMR) from Information Services Division (ISD) Scotland. A validity rate of 84% is quoted from a previous work by the same author [27]. Russ et al. [10] also note the likelihood that SMR datasets will miss some cases of dementia. Stephens et al. [28] highlight the possibility of under-recording in Hospital Episode Statistics (HES), particularly those with earlier or milder forms of dementia. Keenan et al. [29] question the reliability of dementia subtype diagnoses within HES. Those studies describing a validation study or making a comparison with expected rates are shown alongside their respective methodologies in Additional file 5: Table S4.

Brayne et al. [30], Brayne et al. [31] and Clarke et al. [4] made comparisons of prevalence with expected or previously documented rates. As these studies used existing data in part only, it is not possible to draw conclusions regarding the sources of existing data that were used based on these comparisons.

## Discussion

Our systematic review identified 47 articles relevant for inclusion. 36 articles used existing data sources alone for dementia ascertainment, whilst 11 used existing data in conjunction with new data. The existing data sources utilised by the 47 articles included: research databases, death records, clinical notes, national datasets, hospital information systems, radiology records, missing person records, pharmacy records and hospice records. The most commonly used sources were research databases, clinical records and death records. The quality of the description of dementia ascertainment methodology varied widely, with scores from 7 to 16 out of a maximum score of 16. Most studies that completed a validation procedure for dementia ascertainment found that observed rates of dementia were lower than expected.

The initial literature search returned a substantial number of articles in comparison to the number that were included for final analysis. As this review considered a methodology rather than an outcome or specific dementia-related topic, it was necessary to write a broad and inclusive search strategy so as not to miss any relevant articles. As such, it was anticipated that a high proportion of articles would be excluded. The articles included in the final analysis covered a wide variety of specific study topics. The results of the quality measure varied widely, but those papers demonstrating the poorest scores for quality of description were, in nearly all cases, papers in which dementia ascertainment was performed in order to build a cohort for further study [7, 8, 19, 32–34]. This might be expected given that dementia ascertainment was not the focus of these studies and thus the descriptions of method concentrated on other aspects of the studies.

Our assessment of methodology is primarily a narrative account of the sources of existing data utilised in the included articles. The purpose of this review does not include repeating previous extensive literature that compares and comments on diagnostic criteria. The aim is to outline each source and provide some evidence regarding the usefulness or drawbacks of the source.

All of the research databases used by papers in this review rely on the collection of anonymised patient data contributed by participating general practices within the UK. Databases such as the Clinical Practice Research Database (CPRD) have been designed in order to facilitate data-linkage across services, including Hospital Episode Statistics [32]. General practice databases have been used widely in medical research and it has been reported that usage of the General Practice Research Database (GPRD) and Clinical Practice Research Database have resulted in over 800 and 1500 publications respectively [35, 36]. Each database collects data from several hundred general practices and provides records for millions of patients [13, 35, 37]. The volume of data available and the number of general practices involved in such databases indicate a clear benefit to the use of these resources. In order to determine the usefulness of general practice research databases, we must ascertain the validity of diagnostic coding for dementia within the databases. We might consider this in two ways: firstly, do diagnoses contained within the database correlate with information within the general practice records; and secondly, are dementia cases recorded within general practice records an accurate reflection of dementia rates within the population? Using GP questionnaires, Dunn et al. [38] completed a validation study of dementia cases and controls drawn from the GPRD and reported a confirmed diagnosis in 83% of recorded cases. This rate is similar to those reported for Alzheimer’s disease by studies in this review: Imfeld et al. (80%) [11] and Seshadri et al. (83%) [12]. Seshadri et al. did, however, find a much lower validation rate when considering both dementia and Alzheimer’s disease together (48%) [12]. Dunn et al. [38] did not consider dementia prevalence in the study population against previously reported national statistics or alternative databases. In this review, Imfeld et al. [11], Guthrie et al. [16], and Rait et al. [17] all reported lower than expected ascertainment rates from the GPRD, SPICE-PC and THIN database respectively, when compared to previously documented incidence and prevalence. From the findings of the articles included in this review, we would suggest that dementia diagnoses within a general practice research database are not a completely accurate reflection of dementia cases known to the GP or within a population.

A distinct advantage of using death certificates for dementia ascertainment is their availability and the ease of data collection from this source. As dementia is not always the primary cause of death, the inclusion of the diagnosis on the death certificate relies on both the certifying doctor’s familiarity with the patient’s medical history and their opinion as to whether the diagnosis merits inclusion on the certificate. Despite the importance of dementia as a contributory factor or cause of death, rates of reporting on death certificates have historically been poor [39, 40]. A more recent Scottish study did however illustrate an improvement, with 71.5% of deceased patients from a group with known dementia having the diagnosis correctly recorded on their death certificate [41]. In this review, both Doll et al. [42] and Russ et al. [10] demonstrated such under-reporting. It is clear that, despite improvements in diagnosis and reporting, we cannot rely on death certificates to give a completely accurate reflection of dementia cases within a population and studies using this source alone are failing to achieve the best possible ascertainment rates.

The national datasets used by studies in this review include Scottish Morbidity Records (SMR) from the Information Service Division (ISD) of NHS National Services Scotland and Hospital Episode Statistics (HES). SMR are sets of permanently linked datasets, and specifically, SMR01 is a record of inpatient and day-case general hospital admissions, whilst SMR04 is a record of inpatient and day-case psychiatric admissions [43]. HES is a national dataset containing records of all admissions, outpatient appointments and A&E attendances at NHS hospitals in England [44]. In this review, Stephens et al. [28] and Keenan et al. [29] highlight the likelihood of under-recording or inaccuracies in the HES data, but a recent study found that when compared with dementia recording in CPRD and General Practitioner survey responses, HES was accurate in 85% of cases [45]. In order to make assurances regarding the quality of published statistics, ISD Scotland complete regular assessments of collected data. The report published in 2015 found an 89% accuracy for the main diagnosis and a 77.5% accuracy for dementia diagnoses in SMR01 [46]. In our review, Ryan [27] reports a validity rate of 84% based on a previous work. Out-with this review, Russ et al. [47] found that while SMR01 only recorded 53% of known cases of dementia, SMR04 recorded dementia 100% of the time in a cohort of people with known dementia. This would suggest that where a diagnosis of dementia has been made in a psychiatric unit, it is reliably reported within the national dataset SMR04. As most health assessment and treatment in the UK takes place within the National Health Service (NHS) it can be assumed that these datasets are representative of the whole population. They can also be used for large-scale studies. The main drawbacks of these datasets would be that any cases not seen in hospital services would be missed, they rely on cases having been diagnosed, the cases having been diagnosed correctly, and they rely on the diagnoses being recorded in the relevant record systems.

The findings of the study by McGonigal et al. [22] would suggest that psychiatric records and psychiatric case registers are valuable and accurate data sources for pre-senile dementia case ascertainment. It should however be noted that these are historical data, mostly over 30 years old, and admission policies for psychiatric hospitals in Scotland and the UK have changed over that period so this assumption may no longer be tenable. It is possible that a diagnosis, although recorded within clinical records, is simply incorrect. A 2012 Danish study highlighted this issue and in a study of 195 patients registered as having a diagnosis of early onset dementia, the authors found that the diagnosis was correct (according to DSM-IV or ICD-10 criteria) in just 58% of cases [48]. It would therefore seem reasonable to suggest that the most reliable diagnosis taken from written data sources will be where the evidence for diagnostic criteria is present as well as the diagnosis itself.

Studies aiming to evaluate the quality of source information might compare the data collected with information from a second source, for example paper medical records [49]. Concordance between two sources increases the likelihood of correctness, and completeness, but it should be recognised that no source can be assumed to be completely accurate – there is no true “gold-standard” [49]. A diagnosis present in more than one source may superficially appear to be reliable; however, we must consider the possibility that a diagnosis of dementia was initially entered into the notes in error and simply transcribed from one record to another. Between October 2014 and September 2015, the National Patient Safety Agency received almost 99,057 reports relating to failures in documentation from NHS organisations in England and Wales [50].

In using previously collected data for dementia case ascertainment, we are relying on diagnoses having been made and recorded. Using existing data is therefore most effective when diagnostic rates are high. Any population with a poor record for detecting dementia might yield different study results, particularly if undiagnosed cases are associated with particular factors or variables. Regional variation in rates of diagnosis have been reported previously, suggesting that the use of existing data might be more reliable in some geographical areas [51, 52].

All of the sources described by studies included in this review have value, and all are likely to provide ascertainment data for a majority of cases within a population. It would be prudent, however, to be cautious in accepting any documented case as correct without evidence to substantiate the diagnosis. Similarly, if a single source is used, the possibility of missing cases should be considered. We must establish methods for minimising any error, but one should be realistic and accept that any dementia ascertainment method will be open to some error. The main drawback to using existing data of any kind is the potential for undiagnosed cases being missed. For this reason, the most accurate dementia ascertainment process is likely to include prospective follow-up with clinical assessment. Using such methods does however have its own limitations. Collecting prospective data in an ageing population is time-consuming and can lead to delays in the release of findings. This is particularly true if we are to consider influences across the life course or premorbid risk factors. Prospective studies are subject to attrition, due to death or other causes. Using clinical follow-up also restricts the size of a study cohort, with finite funding and resources available for each study. Also significant is the variability of clinical assessment methods across studies, making the comparison of study results less accurate. Within the UK there is an ongoing drive to improve rates of dementia diagnosis and, as such improvements are made, existing data will become increasingly accurate and their use for dementia ascertainment will become increasingly valuable in the study of dementia.

### Considerations for future studies

The evidence for the accuracy of the sources discussed may not be comprehensive and conclusive, but we must attempt to make suggestions for a ‘best possible’ method when performing dementia ascertainment using existing data. In order to minimise any missed cases it would be sensible to collect data from multiple data sources. This might eliminate those cases that have simply failed to be recorded despite a diagnosis having been made. In accessing multiple sources, we may also be more confident that those without a recorded diagnosis are truly dementia free. The most useful method for determining whether any diagnoses are correct would be to consider evidence for a diagnosis within the existing data. Evidence consistent with diagnostic criteria for dementia may not only confirm recorded cases, but identify cases that have failed to be recorded.

When deciding which combination of sources to include in an ascertainment methodology it is useful to consider the advantages and disadvantages of each source, and which combination of sources are likely to yield the highest number of cases. Diagnoses derived from hospital records are of particular value given the high rates of hospital admission for persons with dementia. [53] At a given time-point, it has been estimated that 6% of inpatients in a general hospital have dementia, while 0.6% are aged over 65 and without dementia. [53] National datasets derived from hospital records (such as Hospital Episode Statistics) should contain the same diagnoses as the hospital records themselves. It is however possible that in some situations, such as when there is increased demand on a service, only the main diagnosis is coded. Although the list of diagnoses might be the same, the records could contain further detail to allow for confirmation of the diagnoses. In this sense they may be considered more accurate. The nature of datasets mean that a list of diagnoses, or list of participants with a particular diagnosis can however be made available for a much larger population and in a more time efficient manner. Both, therefore, have their advantages and disadvantages, but using both is unlikely to yield many additional cases. The choice of which to use of the two would depend on the requirements of the study. The similarities, advantages and disadvantages between GP records and GP research databases would be much the same as those described for hospital records. The benefit of GP records over hospital records are that they are more likely to contact records from external services such as social work and housing and contact is likely to be more frequent. These benefits might increase the chance of a diagnosis or symptoms having been recorded. Death certificates have the advantage of being readily available and they are particularly useful as follow-up for participants who do not provide consent for access to records or data linkage. For these reasons, death records would be a useful addition to any other source being used for ascertainment. The disadvantages are that they rely on the physician deeming the diagnosis significant enough to warrant inclusion on the death certificate and they are of no use in identifying dementia cases in the living. Death records and national datasets have the advantage of not being restricted to a specific locality or area, compared with electronic health records that might be held on a different system in each health board. The recording of dementia diagnoses within these sources depends on the proportion of dementia cases identified in the community- in the UK this has previously been shown to be less than 50%. [54].

All of the sources described are likely to identify dementia cases at the more severe end of the spectrum. Regardless of the source, the diagnosis of dementia is more likely to have been made if the condition is more severe and it is therefore more likely to have been recorded. In contrast, early cases are more likely to remain undiagnosed and, as such, would not appear in any existing data. In the case of hospital or GP records or databases, the more severe the condition, the more likely they are to have had contact with a healthcare provider. Similarly, the higher the number of co-morbidities, the more likely they are to have contact with services. This regular contact with services for management of comorbidities may also mean that a diagnosis is more likely to have been made. The more severe the dementia, the more likely it is to be considered a significant factor in cause of death and it is therefore more likely to be recorded on a death certificate.

Clinical assessment is probably the best method for identifying early cases of dementia. There are however problems with non-random screening participation. [55] Early cases are more likely to be recorded in existing data if cases are being identified and diagnosed at an early stage within the community. Diagnoses are in turn more likely to be made if the physician is aware of the condition, appreciates the benefits of diagnosis and is confident in making a diagnosis, or referring for a specialist opinion. Investments in research and public health raise the awareness of dementia among physician, and the general population, meaning that patients are encouraged to present to services rather than accept that changes are merely a consequence of ageing.

### Limitations of the review

Given the variability in the quality of the description of methodology for dementia ascertainment and, in particular, the number that did not provide sufficient information such that an incidence or prevalence rate for dementia could be derived, it was not possible to draw comparisons between the ascertainment rates for different methodologies. A future study considering dementia ascertainment methodologies, using existing data, in incidence and prevalence papers only, might provide the opportunity for direct comparison and an assessment of the effectiveness of different methodologies. It would also be worthwhile for such a study to include studies based out-with the UK. As our study did not, we may have missed ascertainment methodologies that could be replicated using UK sources of existing data. With the use of existing data in dementia studies continuing, it may be worthwhile to consider updating this review in due course. Given that, a single author performed phase one screening of titles and abstracts there is the potential for error. This is, however, unlikely given that the broad search strategy returned a large number of articles that were obviously not relevant to the review.

## Conclusions

In conclusion, our review revealed a lack of consistency with regard to dementia ascertainment methodology using existing data in previous UK studies. Optimising ascertainment is of essential importance in order to increase statistical power, avoid selection bias and enable comparability between studies. We described the benefits of a number of sources of existing data including: death records, national datasets, research databases, and hospital records. Evidence suggested that although each was useful, none was completely accurate when used alone and we would therefore recommend that future studies use a combination of these data sources. Where possible, studies should access records with evidence to confirm, query, or refute the diagnosis. Studies should also calculate a dementia ascertainment rate for the study population to allow for comparison to an expected or previously documented rate. Not only would this help in judging the findings of an individual article, but it would also provide further evidence for guiding dementia ascertainment methodology using existing data.

## Additional files

Additional file 1: MEDLINE search strategy. (DOCX 12 ksb)

Additional file 2: Table S1. Reasons for the exclusion of full-text Articles, by Author. (DOCX 13 kb)

Additional file 3: Table S2. Eligible articles excluded from final review. (DOCX 51 kb)

Additional file 4: Table S3. Quality measure result breakdown. (DOCX 114 kb)

Additional file 5: Table S4. Studies reporting a validation procedure. (DOCX 33 kb)

## Abbreviations

AD: Alzheimer’s disease
CC75C: Cambridge City over-75 s Cohort
CFAS: Medical Research Council Cognitive Function and Ageing Study
CPRD: Clinical Practice Research Database
DSM: Diagnostic and Statistical Manual of Mental Disorders
GPRD: General Practice Research Database
HAA: Hospital Activity Analysis
HES: Hospital Episode Statistics
ICD: International Classification of Diseases
ISD: Information Services Division
Körner: Körner Information System
MHE: Mental Health Enquiry
NHS: National Health Service
NICE: National Institute of Clinical Excellence
NINCDS-ADRDA: National Institute of Neurological and Communicative Disorders and Stroke and the Alzheimer's Disease and Related Disorders Association
NINDS-AIREN: National Institute of Neurological Disorders and Stroke and Association Internationale pour la Recherché et l’Enseignement en Neurosciences
NLSAA: Nottingham Longitudinal Study of Activity and Ageing
SD: Standard deviation
SIGN: Scottish Intercollegiate Guidelines Network
SMR: Scottish Morbidity Records
SPICE-PC: Scottish Programme for Improving Clinical Effectiveness- Primary Care
THIN: The Health Improvement Network
UK: United Kingdom
VD: Vascular dementia
