# Supplementary material for: Dementia ascertainment using existing data in UK longitudinal and cohort studies: a systematic review of methodology
Source: BMC Psychiatry. 2017 Jul 3;17:239. doi: 10.1186/s12888-017-1401-4 (PMC5496178; doi:10.1186/s12888-017-1401-4)
Supplement: Supplementary file 1 — MEDLINE search strategy. (DOCX 12 ksb) [file 12888_2017_1401_MOESM1_ESM.docx]

*Additional File 1: Medline Search Strategy*

**Medline Search Strategy**

1. dementia/ or alzheimer disease/ or aphasia, primary progressive/ or primary progressive nonfluent aphasia/ or dementia, vascular/ or cadasil/ or dementia, multi-infarct/ or diffuse neurofibrillary tangles with calcification/ or frontotemporal lobar degeneration/ or frontotemporal dementia/ or lewy body disease/ or "pick disease of the brain"/

2. dementia*.mp.

3. alzheimer*.mp.

4. "primary progressive aphasia*".mp.

5. "primary progressive non?fluent aphasia*".mp.

6. (vascular adj2 dementia*).mp.

7. cadasil.mp.

8. "multi?infarct dementia".mp.

9. "diffuse neurofibrillary tangles with calcification".mp.

10. "fronto?temporal lobar degeneration".mp.

11. (fronto?temporal adj2 dementia*).mp.

12. ("lewy bod*" adj2 (dementia* or disease*)).mp.

13. (pick* adj2 (dementia* or disease*)).mp.

14. (presenile adj2 dementia*).mp.

15. (senile adj2 dementia*).mp.

16. (semantic adj2 dementia*).mp.

17. 1 or 2 or 3 or 4 or 5 or 6 or 7 or 8 or 9 or 10 or 11 or 12 or 13 or 14 or 15 or 16

18. great britain/ or england/ or northern ireland/ or scotland/ or wales/

19. england.mp.

20. english.mp.

21. scotland.mp.

22. scottish.mp.

23. wales.mp.

24. welsh.mp.

25. "northern ireland".mp.

26. "n ireland".mp.

27. ni.mp.

28. "northern irish".mp.

29. "n irish".mp.

30. gb.mp.

31. gbr.mp.

32. "great britain".mp.

33. britain.mp.

34. british.mp.

35. uk.mp.

36. "united kingdom".mp.

37. 18 or 19 or 20 or 21 or 22 or 23 or 24 or 25 or 26 or 27 or 28 or 29 or 30 or 31 or 32 or 33 or 34 or 35 or 36

38. population surveillance/ or public health surveillance/ or sentinel surveillance/ or cohort studies/ or longitudinal studies/ or follow-up studies/ or prospective studies/ or retrospective studies/

39. cohort*.mp.

40. longitudinal.mp.

41. surveillance.mp.

42. follow?up.mp.

43. prospective.mp.

44. retrospective.mp.

45. 38 or 39 or 40 or 41 or 42 or 43 or 44

46. 17 and 37 and 45
