# Supplementary material for: Dementia ascertainment using existing data in UK longitudinal and cohort studies: a systematic review of methodology
Source: BMC Psychiatry. 2017 Jul 3;17:239. doi: 10.1186/s12888-017-1401-4 (PMC5496178; doi:10.1186/s12888-017-1401-4)
Supplement: Supplementary file 2 — Reasons for the exclusion of full-text Articles, by Author. (DOCX 13 kb) [file 12888_2017_1401_MOESM2_ESM.docx]

*Additional File 2 Table S1: Reasons for the Exclusion of Full-Text Articles, by Author*

| **Reasons for the Exclusion of Full-Text Articles** | |
| --- | --- |
| **First Author (n=297)** | **Second Author (n=297)** |
| a) Not UK (n=28)  b) Non-English language (n=1)  c) Self-referral/ other referral to studies (n=5)  d) Response to census/ survey (n=0)  e) Known neuropathological diagnosis (n=6)  f) Direct referral (n=7)  g) Recruited from hospital, clinics, referrals, other services (n=69)  h) Existing register of cases, study, memory clinic patients, anticholinesterase users or carers (n=23)  i) Dementia not the primary condition of interest (n=7)  j) Animal models of dementia (n=0)  k) Simulated cohorts (n=2)  l) Ascertainment not for dementia (n=1)  m) Not longitudinal or cohort study (n=42)  n) Cases ascertained entirely through baseline and/ or prospective clinical assessment/ new data only (n=61)  o) posters or abstracts (n=21)  p) unclear/ errata/ additional duplicates (n=24) | a) Not UK (n=5)  b) Non-English language (n=1)  c) Self-referral/ other referral to studies (n=0)  d) Response to census/ survey (n=0)  e) Known neuropathological diagnosis (n=0)  f) Direct referral (n=13)  g) Recruited from hospital, clinics, referrals, other services (n=33)  h) Existing register of cases, study, memory clinic patients, anticholinesterase users or carers (n=41)  i) Dementia not the primary condition of interest (n=9)  j) Animal models of dementia (n=0)  k) Simulated cohorts (n=0)  l) Ascertainment not for dementia (n=3)  m) Not longitudinal or cohort study (n=83)  n) Cases ascertained entirely through baseline and/ or prospective clinical assessment/ new data only (n=42)  o) posters or abstracts (n=44)  p) unclear/ errata/ additional duplicates (n=23) |
