# Supplementary material for: Dementia ascertainment using existing data in UK longitudinal and cohort studies: a systematic review of methodology
Source: BMC Psychiatry. 2017 Jul 3;17:239. doi: 10.1186/s12888-017-1401-4 (PMC5496178; doi:10.1186/s12888-017-1401-4)
Supplement: Supplementary file 3 — Eligible articles excluded from final review. (DOCX 51 kb) [file 12888_2017_1401_MOESM3_ESM.docx]

*Additional File 3 Table S2: Eligible Articles Excluded from Final Review*

| **GROUP** | **INCLUDED/ EXCLUDED** | **AUTHOR** | **STUDY TOPIC** | **DEMENTIA OUTCOME OR COHORT^†^** | **EXISTING DATA USE** | **DATA SOURCES** | **QUALITY SCORE** |
| --- | --- | --- | --- | --- | --- | --- | --- |
| 1 | Included | Brayne et al. [1] | Dementia at death and prevention | Outcome | Part | Death certificates | 15 |
| 1 | Excluded | Nicoll et al. [2] | Association between APOE, neuropathology and dementia | Outcome | Part | Death certificates | 11 |
| 1 | Excluded | Valenzuela et al. [3] | Cognitive lifestyle and protection from dementia | Outcome | Part | Death certificates | 13 |
| 1 | Excluded | Wharton et al. [4] | Epidemiological neuropathology | Outcome | Part | Death certificates | 7 |
| 2 | Included | Brayne et al. [5] | Neuropathological correlates of dementia | Outcome | Part | Death certificates | 15 |
| 2 | Excluded | Perales et al. [6] | Health related quality of life | Cohort | Part | Death certificates | 13 |
| 3 | Included | Clarke et al. [7] | Dementia incidence | Outcome | Part | Death certificates, hospital case notes | 14 |
| 3 | Excluded | Morgan et al. [8] | Incidence of dementia | Outcome | Part | Death certificates, hospital case notes | 13 |
| 3 | Excluded | Morgan et al. [9] | Incidence of dementia | Outcome | Part | Death certificates, hospital case notes | 13 |
| 3 | Excluded | Morgan et al. [10] | Risk factors for incident dementia | Outcome | Part | Death certificates, hospital case notes | 12 |
| 4 | Included | Imfeld et al. [11] | Epidemiology, comorbidities and medication in AD & VD | Outcome | Full | GPRD* | 9 |
| 4 | Excluded | Imfeld et al. [12] | Risk of stroke in AD or VD | Cohort | Full | GPRD* | 8 |
| 4 | Excluded | Imfeld et al. [13] | Seizures in AD or VD | Cohort | Full | GPRD* | 8 |
| 5 | Included | Newens et al. [14] | Ascertainment, incidence, prevalence & survival in pre-senile AD | Outcome | Part | Electronic hospital information systems (^₸^HAA, MHE, Körner), neuroradiology records, hospital case notes | 13 |
| 5 | Excluded | Kay et al. [15] | Survival, place of death and death certification in pre-senile dementia | Outcome | Part | Electronic hospital information systems (^₸^HAA, MHE, Körner), neuroradiology records, hospital case notes | 12 |
| 5 | Excluded | Newens et al. [16] | Death certification after a diagnosis of pre-senile dementia | Cohort | Part | Electronic hospital information systems (^₸^HAA, MHE, Körner), neuroradiology records, case notes | 8 |
| 6 | Included | Keenan et al. [17] | Glaucoma and AD/ VD | Both | Full | Hospital Episode Statistics | 15 |
| 6 | Excluded | Keenan et al. [18] | Macular degeneration, AD and dementia | Both | Full | Hospital Episode Statistics | 14 |
| 7 | Included | Wotton et al. [19] | Obesity and subsequent dementia | Outcome | Full | Hospital Episode Statistics linked with death records | 14 |
| 7 | Excluded | Goldacre et al. [20] | Cataract surgery and dementia | Cohort | Full | Hospital Episodes Statistics linked with mortality records | 8 |
| 7 | Excluded | Smolina et al. [21] | Risk of dementia in diabetes | Outcome | Full | Hospital Episode Statistics, linked to death records | 13 |
| 8 | Included | Sorahan et al. [22] | AD, MND and PD in magnetic field exposure | Outcome | Full | Death records | 12 |
| 8 | Excluded | Sorahan et al. [23] | Neurodegenerative disease and magnetic field exposure | Outcome | Full | Death records | 10 |
| 9 | Included |  |  |  |  |  |  |
| 9 | Excluded | Staff et al. [24] | Brain volume and survival | Outcome | Part | Case notes, imaging results | 15 |

*^†^: Was dementia ascertainment performed as the study outcome, or was dementia ascertainment performed in order to form a dementia cohort for which another outcome was determined.*

*^₸^HAA: Hospital Activity Analysis; MHE: Mental Health Enquiry system; Körner: Korner Episode Statstics (hospital information systems).*

**GPRD: General Practice Research Database*

**References**

1. Brayne C, Gao L, Dewey M, Matthews FE, Medical Research Council Cognitive F, Ageing Study I: **Dementia before death in ageing societies--the promise of prevention and the reality**. *PLoS Medicine / Public Library of Science* 2006, **3**(10):e397.

2. Nicoll JAR, Savva GM, Stewart J, Matthews FE, Brayne C, Ince P, Med Res Council Cognitive F: **Association between APOE genotype, neuropathology and dementia in the older population of England and Wales**. *Neuropathology and Applied Neurobiology* 2011, **37**(3):285-294.

3. Valenzuela MJ, Matthews FE, Brayne C, Ince P, Halliday G, Kril JJ, Dalton MA, Richardson K, Forster G, Sachdev PS *et al*: **Multiple biological pathways link cognitive lifestyle to protection from dementia**. *Biological Psychiatry* 2012, **71**(9):783-791.

4. Wharton SB, Brayne C, Savva GM, Matthews FE, Forster G, Simpson J, Lace G, Ince PG, Med Res Council Cognitive F: **Epidemiological Neuropathology: The MRC Cognitive Function and Aging Study Experience**. *Journal of Alzheimers Disease* 2011, **25**(2):359-372.

5. Brayne C, Richardson K, Matthews FE, Fleming J, Hunter S, Xuereb JH, Paykeld E, Mukaetova-Ladinska EB, Huppert FA, O'Sullivan A *et al*: **Neuropathological correlates of dementia in over-80-year-old brain donors from the population-based Cambridge City over-75s Cohort (CC75C) study**. *Journal of Alzheimer's Disease* 2009, **18**(3):645-658.

6. Perales J, Cosco TD, Stephan BC, Fleming J, Martin S, Haro JM, Brayne C, Study CC: **Health-related quality of life in the Cambridge City over-75s Cohort (CC75C): development of a dementia-specific scale and descriptive analyses**. *BMC geriatrics* 2014, **14**:18.

7. Clarke D, Morgan K, Lilley J, Arie T, Jones R, Waite J, Prettyman R: **Dementia and 'borderline dementia' in Britain: 8-year incidence and post-screening outcomes**. *Psychological Medicine* 1996, **26**(4):829-835.

8. Morgan K, Lilley J, Arie T, Byrne J, Jones R, Waite J: **Incidence of dementia: preliminary findings from the Nottingham Longitudinal Study of Activity and Ageing**. *Neuroepidemiology* 1992, **11 Suppl 1**:80-83.

9. Morgan K, Lilley JM, Arie T, Byrne EJ, Jones R, Waite J: **Incidence of dementia in a representative British sample**. *British Journal of Psychiatry* 1993, **163**:467-470.

10. Morgan K, Lilley JM: **RISK-FACTORS AMONG INCIDENT CASES OF DEMENTIA IN A REPRESENTATIVE BRITISH SAMPLE**. *International Journal of Geriatric Psychiatry* 1994, **9**(1):11-15.

11. Imfeld P, Pernus YBB, Jick SS, Meier CR: **Epidemiology, Co-Morbidities, and Medication Use of Patients with Alzheimer's Disease or Vascular Dementia in the UK**. *Journal of Alzheimers Disease* 2013, **35**(3):565-573.

12. Imfeld P, Bodmer M, Schuerch M, Jick SS, Meier CR: **Risk of incident stroke in patients with Alzheimer disease or vascular dementia**. *Neurology* 2013, **81**(10):910-919.

13. Imfeld P, Bodmer M, Schuerch M, Jick SS, Meier CR: **Seizures in patients with Alzheimer's disease or vascular dementia: A population-based nested case-control analysis**. *Epilepsia* 2013, **54**(4):700-707.

14. Newens AJ, Forster DP, Kay LDWK, Kirkup W, Bates D, Edwardson J: **Clinically diagnosed presenile dementia of the Alzheimer type in the Northern Health Region: Ascertainment, prevalence, incidence and survival**. *Psychological Medicine* 1993, **23**(3):631-644.

15. Kay DW, Forster DP, Newens AJ: **Long-term survival, place of death, and death certification in clinically diagnosed pre-senile dementia in northern England: Follow-up after 8-12 years**. *The British Journal of Psychiatry* 2000, **177**:156-162.

16. Newens AJ, Forster DP, Kay DW: **Death certification after a diagnosis of presenile dementia**. *Journal of Epidemiology & Community Health* 1993, **47**(4):293-297.

17. Keenan TDL, Goldacre R, Goldacre MJ: **Associations between primary open angle glaucoma, Alzheimer's disease and vascular dementia: Record linkage study**. *British Journal of Ophthalmology* 2015, **99**(4):524-527.

18. Keenan TDL, Goldacre R, Goldacre MJ: **Associations Between Age-Related Macular Degeneration, Alzheimer Disease, and Dementia: Record Linkage Study of Hospital Admissions**. *Jama Ophthalmology* 2014, **132**(1):63-68.

19. Wotton CJ, Goldacre MJ: **Age at obesity and association with subsequent dementia: Record linkage study**. *Postgraduate Medical Journal* 2014, **90**(1068):547-551.

20. Goldacre R, Yeates D, Goldacre MJ, Keenan TDL: **Cataract surgery in people with dementia: An English National Record linkage study**. *Journal of the American Geriatrics Society* 2015, **63**(9):1953-1955.

21. Smolina K, Wotton CJ, Goldacre MJ: **Risk of dementia in patients hospitalised with type 1 and type 2 diabetes in England, 1998-2011: a retrospective national record linkage cohort study**. *Diabetologia* 2015, **58**(5):942-950.

22. Sorahan T, Kheifets L: **Mortality from Alzheimer's, motor neuron and Parkinson's disease in relation to magnetic field exposure: Findings from the study of UK electricity generation and transmission workers, 1973-2004**. *Occupational and Environmental Medicine* 2007, **64**(12):820-826.

23. Sorahan T, Mohammed N: **Neurodegenerative disease and magnetic field exposure in UK electricity supply workers**. *Occupational medicine (Oxford, England)* 2014, **64**(6):454-460.

24. Staff RT, Murray AD, Ahearn T, Salarirad S, Mowat D, Starr JM, Deary IJ, Lemmon H, Whalley LJ: **Brain volume and survival from age 78 to 85: the contribution of Alzheimer-type magnetic resonance imaging findings**. *Journal of the American Geriatrics Society* 2010, **58**(4):688-695.
