# Supplementary material for: Dementia ascertainment using existing data in UK longitudinal and cohort studies: a systematic review of methodology
Source: BMC Psychiatry. 2017 Jul 3;17:239. doi: 10.1186/s12888-017-1401-4 (PMC5496178; doi:10.1186/s12888-017-1401-4)
Supplement: Supplementary file 4 — Quality measure result breakdown. (DOCX 114 kb) [file 12888_2017_1401_MOESM4_ESM.docx]

*Additional File 4 Table S3: Quality Measure Result Breakdown*

|  | **1** | **2** | **3** | **4** | **5** | **6** | **7** | **Total Score** |
| --- | --- | --- | --- | --- | --- | --- | --- | --- |
| Baker et al. [1] | C | BII | B | A | AI | B | A | 9 |
| Brayne et al. [2] | A | AII | A | A | AI | A | A | 15 |
| Brayne et al. [3] | A | AII | A | A | AI | A | A | 15 |
| Chen et al. [4] | C | BII | B | A | AII | B | A | 8 |
| Clarke et al. [5] | A | AII | A | A | AII | A | A | 14 |
| Cook et al. [6] | C | BII | B | A | AI | B | A | 9 |
| Crugel et al. [7] | D | C | B | B | AI | A | A | 7 |
| Doll et al. [8] | A | C | A | A | AII | A | A | 11 |
| Dregan et al. [9] | A | AII | A | A | AII | B | A | 13 |
| Goh et al. [10] | A | AII | A | A | AI | B | A | 14 |
| Goldacre et al. [11] | D | BII | B | B | AI | A | A | 8 |
| Grant et al. [12] | A | AI | B | A | AI | B | A | 14 |
| Guthrie et al. [13] | A | AII | B | A | AI | A | A | 14 |
| Heath et al. [14] | A | AI | A | A | AI | A | A | 16 |
| Houttekier et al. [15] | A | AII | B | A | AI | B | A | 13 |
| Imfeld et al. [16] | C | BII | B | A | AI | B | A | 8 |
| Imfeld et al. [17] | C | BII | B | A | AI | B | A | 8 |
| Imfeld et al. [18] | C | BII | B | A | AI | A | A | 9 |
| Karlinsky et al. [19] | B | AII | B | A | AII | B | B | 10 |
| Kay et al. [20] | B | AI | B | B | AII | A | A | 12 |
| Keenan et al. [21] | A | AII | A | A | AII | A | A | 14 |
| Keenan et al. [22] | A | AII | A | A | AI | A | A | 15 |
| Kehoe et al. [23] | C | BII | B | A | AI | B | A | 9 |
| Lu et al. [24] | A | AII | A | A | AI | B | A | 14 |
| Martinez et al. [25] | C | BII | B | A | AI | B | A | 9 |
| McCarthy et al. [26] | C | BII | B | A | AII | B | A | 8 |
| McGonigal et al. [27] | B | AI | B | B | AI | A | A | 13 |
| Morgan et al. [28] | A | AI | A | A | AI | B | A | 15 |
| Morgan et al. [29] | A | AII | A | A | AII | B | A | 13 |
| Morgan et al. [30] | A | AII | A | A | AII | B | A | 13 |
| Morgan et al. [31] | A | AII | B | A | AII | B | A | 12 |
| Newens et al. [32] | B | AI | B | B | AI | A | A | 13 |
| Newens et al. [33] | D | BII | B | B | AI | A | A | 8 |
| Nicoll et al. [34] | A | AII | A | A | B | B | B | 11 |
| Palmer et al. [35] | C | BI | B | B | B | B | A | 7 |
| Pendlebury et al. [36] | A | AII | A | A | AI | B | A | 14 |
| Perales et al. [37] | A | AII | B | A | AI | B | A | 13 |
| Perera et al. [38] | C | C | B | A | AI | B | A | 8 |
| Qizilbash et al. [39] | A | AII | A | A | AII | B | A | 13 |
| Rait et al. [40] | C | BII | B | A | AI | A | A | 10 |
| Renvoize et al. [41] | B | AI | A | B | AII | A | A | 13 |
| Reyniers et al. [42] | A | BII | B | B | AI | B | A | 10 |
| Russ et al. [43] | A | AI | A | A | AI | A | A | 16 |
| Ryan [44] | A | AII | B | A | AI | A | A | 14 |
| Sampson et al. [45] | A | AII | A | B | AI | B | A | 13 |
| Seshadri et al. [46] | A | AII | A | A | AI | B | A | 14 |
| Shah et al. [47] | A | AI | B | A | AII | B | A | 12 |
| Sleeman et al. [48] | C | BII | B | B | AI | B | A | 8 |
| Smolina et al. [49] | A | AII | B | A | AI | B | A | 13 |
| Sorahan et al. [50] | A | C | A | A | AI | A | A | 12 |
| Sorahan et al. [51] | A | C | A | B | AI | B | A | 10 |
| Staff et al. [52] | A | AI | A | A | AI | B | A | 15 |
| Stephens et al. [53] | B | BII | B | B | AI | B | A | 9 |
| Su et al. [54] | A | AII | A | A | AI | B | A | 14 |
| Valenzuela et al. [55] | A | AII | A | A | AII | B | A | 13 |
| De Vries et al. [56] | A | AII | B | A | AII | B | A | 12 |
| Whalley et al. [57] | A | AI | A | A | AI | B | A | 15 |
| Whalley et al. [58] | A | AI | B | A | AI | B | A | 14 |
| Wharton et al. [59] | A | C | B | A | B | C | A | 7 |
| White et al. [60] | A | BII | B | A | AII | A | A | 11 |
| Wilcock et al. [61] | A | BII | B | A | AII | B | A | 10 |
| Woodburn et al. [62] | B | AI | B | B | AI | B | A | 12 |
| Wotton et al. [63] | A | AII | A | A | AI | B | A | 14 |

**References**

1. Baker NL, Cook MN, Arrighi HM, Bullock R. **Hip fracture risk and subsequent mortality among Alzheimer's disease patients in the United Kingdom, 1988-2007**. *Age & Ageing* 2011, **40**(1):49-54.

2. Brayne C, Gao L, Dewey M, Matthews FE, Medical Research Council Cognitive Function and Ageing Study I. **Dementia before death in ageing societies--the promise of prevention and the reality**. *PLoS Medicine / Public Library of Science* 2006, **3**(10):e397.

3. Brayne C, Richardson K, Matthews FE, Fleming J, Hunter S, Xuereb JH, Paykel E, Mukaetova-Ladinska EB, Huppert FA, O'Sullivan A *et al*. **Neuropathological Correlates of Dementia in Over-80-Year-Old Brain Donors from the Population-Based Cambridge City over-75s Cohort (CC75C) Study**. *Journal of Alzheimers Disease* 2009, **18**(3):645-658.

4. Chen L, Reed C, Happich M, Nyhuis A, Lenox-Smith A. **Health care resource utilisation in primary care prior to and after a diagnosis of Alzheimer's disease: a retrospective, matched case-control study in the United Kingdom**. *BMC geriatrics* 2014, **14**:76.

5. Clarke D, Morgan K, Lilley J, Arie T, Jones R, Waite J, Prettyman R. **Dementia and 'borderline dementia' in Britain: 8-year incidence and post-screening outcomes**. *Psychological Medicine* 1996, **26**(4):829-835.

6. Cook M, Baker N, Lanes S, Bullock R, Wentworth C, Michael Arrighi H. **Incidence of stroke and seizure in Alzheimer's disease dementia**. *Age and Ageing* 2015, **44**(4):695-699.

7. Crugel M, Paton G, Singh P, Jeboda R, Treloar A. **Antipsychotics in people with dementia: Frequency of use and rationale for prescribing in a UK mental health service**. *Psychiatrist* 2012, **36**(5):165-169.

8. Doll R, Peto R, Boreham J, Sutherland I. **Smoking and dementia in male British doctors: prospective study**. *BMJ* 2000, **320**(7242):1097-1102.

9. Dregan A, Chowienczyk P, Gulliford MC. **Are Inflammation and Related Therapy Associated with All-Cause Dementia in a Primary Care Population?** *Journal of Alzheimer's Disease* 2015, **46**(4):1039-1047.

10. Goh KL, Bhaskaran K, Minassian C, Evans SJW, Smeeth L, Douglas IJ. **Angiotensin receptor blockers and risk of dementia: Cohort study in UK Clinical Practice Research Datalink**. *British Journal of Clinical Pharmacology* 2015, **79**(2):337-350.

11. Goldacre R, Yeates D, Goldacre MJ, Keenan TDL. **Cataract surgery in people with dementia: An English National Record linkage study**. *Journal of the American Geriatrics Society* 2015, **63**(9):1953-1955.

12. Grant RL, Drennan VM, Rait G, Petersen I, Iliffe S. **First Diagnosis and Management of Incontinence in Older People with and without Dementia in Primary Care: A Cohort Study Using The Health Improvement Network Primary Care Database**. *Plos Medicine* 2013, **10**(8).

13. Guthrie B, Clark SA, McCowan C. **The burden of psychotropic drug prescribingin people with dementia: a populationdatabase study**. *Age & Ageing* 2010, **39**(5):637-642.

14. Heath CA, Mercer SW, Guthrie B. **Vascular comorbidities in younger people with dementia: a cross-sectional population-based study of 616 245 middle-aged people in Scotland**. *Journal of Neurology Neurosurgery and Psychiatry* 2015, **86**(9):959-964.

15. Houttekier D, Cohen J, Bilsen J, Addington-Hall J, Onwuteaka-Philipsen BD, Deliens L. **Place of Death of Older Persons with Dementia. A Study in Five European Countries**. *Journal of the American Geriatrics Society* 2010, **58**(4):751-756.

16. Imfeld P, Bodmer M, Schuerch M, Jick SS, Meier CR. **Risk of incident stroke in patients with Alzheimer disease or vascular dementia**. *Neurology* 2013, **81**(10):910-919.

17. Imfeld P, Bodmer M, Schuerch M, Jick SS, Meier CR. **Seizures in patients with Alzheimer's disease or vascular dementia: a population-based nested case-control analysis**. *Epilepsia* 2013, **54**(4):700-707.

18. Imfeld P, Pernus YBB, Jick SS, Meier CR. **Epidemiology, Co-Morbidities, and Medication Use of Patients with Alzheimer's Disease or Vascular Dementia in the UK**. *Journal of Alzheimers Disease* 2013, **35**(3):565-573.

19. Karlinsky H, Macdonald AM, Berg JM. **Primary degenerative dementia of the Alzheimer type in twins: Initial findings from the Maudsley Hospital Twin Register**. *International Journal of Geriatric Psychiatry* 1992, **7**(8):603-610.

20. Kay DW, Forster DP, Newens AJ. **Long-term survival, place of death, and death certification in clinically diagnosed pre-senile dementia in northern England: Follow-up after 8-12 years**. *The British Journal of Psychiatry* 2000, **177**:156-162.

21. Keenan TDL, Goldacre R, Goldacre MJ. **Associations Between Age-Related Macular Degeneration, Alzheimer Disease, and Dementia: Record Linkage Study of Hospital Admissions**. *Jama Ophthalmology* 2014, **132**(1):63-68.

22. Keenan TDL, Goldacre R, Goldacre MJ. **Associations between primary open angle glaucoma, Alzheimer's disease and vascular dementia: Record linkage study**. *British Journal of Ophthalmology* 2015, **99**(4):524-527.

23. Kehoe PG, Davies NM, Martin RM, Ben-Shlomo Y. **Associations of angiotensin targeting antihypertensive drugs with mortality and hospitalization in primary care patients with dementia**. *Journal of Alzheimer's Disease* 2013, **33**(4):999-1008.

24. Lu N, Zhang Y, Ascherio A, Hernan M, Neogi T, Dubreuil M, Choi H. **Gout and the risk of alzheimer's disease: A population-based cohort study**. *Arthritis and Rheumatology* 2014, **66**:S364.

25. Martinez C, Jones RW, Rietbrock S. **Trends in the prevalence of antipsychotic drug use among patients with Alzheimer's disease and other dementias including those treated with antidementia drugs in the community in the UK: a cohort study**. *Bmj Open* 2013, **3**(1).

26. McCarthy M, AddingtonHall J, Altmann D. **The experience of dying with dementia: A retrospective study**. *International Journal of Geriatric Psychiatry* 1997, **12**(3):404-409.

27. McGonigal G, Thomas B, McQuade C, Starr JM, MacLennan WJ, Whalley LJ. **Epidemiology of Alzheimer's presenile dementia in Scotland, 1974-88**. *BMJ* 1993, **306**(6879):680-683.

28. Morgan GS, Gallacher J, Bayer A, Fish M, Ebrahim S, Ben-Shlomo Y. **Physical activity in middle-age and dementia in later life: findings from a prospective cohort of men in Caerphilly, South Wales and a meta-analysis**. *Journal of Alzheimer's Disease* 2012, **31**(3):569-580.

29. Morgan K, Lilley J, Arie T, Byrne J, Jones R, Waite J. **Incidence of dementia: preliminary findings from the Nottingham Longitudinal Study of Activity and Ageing**. *Neuroepidemiology* 1992, **11 Suppl 1**:80-83.

30. Morgan K, Lilley JM, Arie T, Byrne EJ, Jones R, Waite J. **Incidence of dementia in a representative British sample**. *British Journal of Psychiatry* 1993, **163**:467-470.

31. Morgan K, Lilley JM. **Risk-Factors Among Incident Cases of Dementia in a Representative British Sample**. *International Journal of Geriatric Psychiatry* 1994, **9**(1):11-15.

32. Newens AJ, Forster DP, Kay LDWK, Kirkup W, Bates D, Edwardson J. **Clinically diagnosed presenile dementia of the Alzheimer type in the Northern Health Region: Ascertainment, prevalence, incidence and survival**. *Psychological Medicine* 1993, **23**(3):631-644.

33. Newens AJ, Forster DP, Kay DW. **Death certification after a diagnosis of presenile dementia**. *Journal of Epidemiology & Community Health* 1993, **47**(4):293-297.

34. Nicoll JAR, Savva GM, Stewart J, Matthews FE, Brayne C, Ince P, Medical Research Council Cognitive Function and Ageing Study. **Association between APOE genotype, neuropathology and dementia in the older population of England and Wales**. *Neuropathology and Applied Neurobiology* 2011, **37**(3):285-294.

35. Palmer K, Inskip H, Martyn C, Coggon D. **Dementia and occupational exposure to organic solvents**. *Occupational & Environmental Medicine* 1998, **55**(10):712-715.

36. Pendlebury ST, Chen PJ, Welch SJ, Cuthbertson FC, Wharton RM, Mehta Z, Rothwell PM, Oxford Vascular Study. **Methodological Factors in Determining Risk of Dementia After Transient Ischemic Attack and Stroke: (II) Effect of Attrition on Follow-Up**. *Stroke* 2015, **46**(6):1494-1500.

37. Perales J, Cosco TD, Stephan BC, Fleming J, Martin S, Haro JM, Brayne C, Study CC75C. **Health-related quality of life in the Cambridge City over-75s Cohort (CC75C): development of a dementia-specific scale and descriptive analyses**. *BMC geriatrics* 2014, **14**:18.

38. Perera G, Khondoker M, Broadbent M, Breen G, Stewart R. **Factors associated with response to acetylcholinesterase inhibition in dementia: A cohort study from a secondary mental health care case register in London**. *PLoS ONE* 2014, **9 (11) (no pagination)**(e109484).

39. Qizilbash N, Gregson J, Johnson ME, Pearce N, Douglas I, Wing K, Evans SJ, Pocock SJ. **BMI and risk of dementia in two million people over two decades: a retrospective cohort study**. *Lancet Diabetes Endocrinol* 2015, **3**.

40. Rait G, Walters K, Bottomley C, Petersen I, Iliffe S, Nazareth I. **Survival of people with clinical diagnosis of dementia in primary care: cohort study**. *BMJ* 2010, **341**:c3584.

41. Renvoize E, Hanson M, Dale M. **Prevalence and causes of young onset dementia in an English health district**. *International Journal of Geriatric Psychiatry* 2011, **26**(1):106-107.

42. Reyniers T, Deliens L, Pasman HR, Morin L, Addington-Hall J, Frova L, Cardenas-Turanzas M, Onwuteaka-Philipsen B, Naylor W, Ruiz-Ramos M *et al*. **International Variation in Place of Death of Older People Who Died From Dementia in 14 European and non-European Countries**. *Journal of the American Medical Directors Association* 2015, **16**(2):165-171.

43. Russ TC, Gatz M, Pedersen NL, Hannah J, Wyper G, Batty GD, Deary IJ, Starr JM. **Geographical variation in dementia: Examining the role of environmental factors in Sweden and Scotland**. *Epidemiology* 2015, **26**(2):263-270.

44. Ryan DH. **Age-specific hospital incidence rates in dementia**. *Dementia* 1994, **5**(1):29-35.

45. Sampson EL, Gould V, Lee D, Blanchard MR. **Differences in care received by patients with and without dementia who died during acute hospital admission: a retrospective case note study**. *Age & Ageing* 2006, **35**(2):187-189.

46. Seshadri S, Zornberg GL, Derby LE, Myers MW, Jick H, Drachman DA. **Postmenopausal estrogen replacement therapy and the risk of Alzheimer disease**. *Archives of Neurology* 2001, **58**(3):435-440.

47. Shah SM, Carey IM, Harris T, DeWilde S, Cook DG. **The impact of dementia on influenza vaccination uptake in community and care home residents**. *Age & Ageing* 2012, **41**(1):64-69.

48. Sleeman KE, Ho YK, Verne J, Gao W, Higginson IJ, GUIDE_Care project. **Reversal of English trend towards hospital death in dementia: a population-based study of place of death and associated individual and regional factors, 2001-2010**. *BMC Neurology* 2014, **14**:59.

49. Smolina K, Wotton CJ, Goldacre MJ. **Risk of dementia in patients hospitalised with type 1 and type 2 diabetes in England, 1998-2011: a retrospective national record linkage cohort study**. *Diabetologia* 2015, **58**(5):942-950.

50. Sorahan T, Kheifets L. **Mortality from Alzheimer's, motor neuron and Parkinson's disease in relation to magnetic field exposure: findings from the study of UK electricity generation and transmission workers, 1973-2004**. *Occupational & Environmental Medicine* 2007, **64**(12):820-826.

51. Sorahan T, Mohammed N. **Neurodegenerative disease and magnetic field exposure in UK electricity supply workers**. *Occupational medicine (Oxford, England)* 2014, **64**(6):454-460.

52. Staff RT, Murray AD, Ahearn T, Salarirad S, Mowat D, Starr JM, Deary IJ, Lemmon H, Whalley LJ. **Brain volume and survival from age 78 to 85: the contribution of Alzheimer-type magnetic resonance imaging findings**. *Journal of the American Geriatrics Society* 2010, **58**(4):688-695.

53. Stephens P, Chikh K, Leufkens H. **Prescribing of antipsychotics in people with dementia in acute general hospitals in England: 2010-2012**. *European Geriatric Medicine* 2014, **5**(6):394-398.

54. Su YP, Chang CK, Hayes RD, Perera G, Broadbent M, To D, Hotopf M, Stewart R. **Mini-mental state examination as a predictor of mortality among older people referred to secondary mental healthcare**. *PLoS ONE* 2014, **9 (9) (no pagination)**(e105312).

55. Valenzuela MJ, Matthews FE, Brayne C, Ince P, Halliday G, Kril JJ, Dalton MA, Richardson K, Forster G, Sachdev PS *et al.* **Multiple biological pathways link cognitive lifestyle to protection from dementia**. *Biological Psychiatry* 2012, **71**(9):783-791.

56. Vries K, Nowell A. **Dementia deaths in hospice: a retrospective case note audit**. *International Journal of Palliative Nursing* 2011, **17**(12):581-585.

57. Whalley LJ, Sharma S, Fox HC, Murray AD, Staff RT, Duthie AC, Deary IJ, Starr JM. **Anticholinergic drugs in late life: adverse effects on cognition but not on progress to dementia**. *Journal of Alzheimer's Disease* 2012, **30**(2):253-261.

58. Whalley LJ, Starr JM, Athawes R, Hunter D, Pattie A, Deary IJ. **Childhood mental ability and dementia**. *Neurology* 2000, **55**(10):1455-1459.

59. Wharton SB, Brayne C, Savva GM, Matthews FE, Forster G, Simpson J, Lace G, Ince PG, Medical Research Council Cognitive Function and Ageing Study. **Epidemiological Neuropathology: The MRC Cognitive Function and Aging Study Experience**. *Journal of Alzheimers Disease* 2011, **25**(2):359-372.

60. White EB, Montgomery P. **Dementia, walking outdoors and getting lost: incidence, risk factors and consequences from dementia-related police missing-person reports**. *Aging & Mental Health* 2015, **19**(3):224-230.

61. Wilcock J, Iliffe S, Griffin M, Jain P, Thune-Boyle I, Lefford F, Rapp D. **Tailored educational intervention for primary care to improve the management of dementia: The EVIDEM-ED cluster randomized controlled trial**. *Trials* 2013, **14 (1) (no pagination)**(397).

62. Woodburn KJ, Johnstone EC. **Early-onset dementia in Lothian, Scotland: an analysis of clinical features and patterns of decline**. *Health Bulletin* 1999, **57**(6):384-392.

63. Wotton CJ, Goldacre MJ. **Age at obesity and association with subsequent dementia: Record linkage study**. *Postgraduate Medical Journal* 2014, **90**(1068):547-551.
