# Supplementary material for: Dementia ascertainment using existing data in UK longitudinal and cohort studies: a systematic review of methodology
Source: BMC Psychiatry. 2017 Jul 3;17:239. doi: 10.1186/s12888-017-1401-4 (PMC5496178; doi:10.1186/s12888-017-1401-4)
Supplement: Supplementary file 5 — Studies reporting a validation procedure. (DOCX 33 kb) [file 12888_2017_1401_MOESM5_ESM.docx]

| **Article** | **Source/ Methodology** | **Validation/ Comparison Result** |
| --- | --- | --- |
| Doll et al.[1] | Death Certificates:  Dementia ‘mentioned’ on death certificate. | -30% of dementia cases recorded on death certificates when compared to EURODEM statistics |
| Guthrie et al.[2] | *SPICE-PC:  Read codes for dementia,  OR  Prescription of acetylcholinesterase inhibitor. | -prevalence approximately half of expected |
| Heath et al.[3] | Unnamed general practice research database:  Read codes for dementia,  OR  Prescription of acetylcholinesterase inhibitor. | -prevalence was close to middle of expected range |
| Imfeld et al.[4] | *GPRD:  Stage I- read code for dementia, OR, prescription for acetylcholinesterase inhibitor.  Stage II- algorithm for AD or VD (based on DSM-IV, NINCDS-ADRDA, NINDS-AIREN, NICE & SIGN). | -80% AD cases and 75% VD cases confirmed by GP questionnaire  -incidence rates of AD 3-6 times lower than previous studies |
| McGonigal et al.[5] | ^‡^ISD Scotland Data for psychiatric hospitals & hospital records:  Stage I: diagnostic codes for dementia in SMR,  Stage II: NINCDS-ADRDA criteria and Hachinski score applied to records. | -97% of participants with pre-senile dementia were cared for within psychiatric services  -annual incidence of presenile dementia determined using hospital records comparable to annual incidence rates quoted by a national study. |
| Newens et al.[6] | Electronic hospital information systems:  Stage I: potential cases identified by ICD-9 codes from information systems, referrals for CT with dementing process, (*and contact with services*).  Stage II: case notes for all examined for DSM-III-R criteria for dementia, then algorithm for pre-senile AD | -prevalence rate similar to rates documented elsewhere |
| Rait et al.[7] | *THIN:  Read codes for dementia. | -incidence rates significantly lower than expected when compared with EURODEM and CFAS studies |
| Renvoize et al.[8] | Computerised medical and social records:  Stage I: discharge diagnosis of dementia in computer system,  Stage II: “criteria for dementia” from notes (criteria not specified) | -prevalence rate found to be consistent with previous studies |
| Russ et al.[9] | ISD Scotland data, death certificates, records for a nursing home medical practice:  ICD-9 & 10 codes for dementia from SMR data and death certificates, and dementia status reported by a medical practice. | -when compared to multiple sources, death certificates missed 16-18% of cases  -general practice records did not identify all cases identified by record linkage |
| Ryan et al.[10] | ^‡^ISD Scotland data:  ICD-8 & 9 codes for dementia. | -validity rate of 84% quoted from a previous work by the same author |
| Seshadri et al.[11] | *GPRD, GP records:  Stage I: computer diagnosis of dementia,  Stage II: records for each reviewed to confirm diagnosis based on NINCDS-ADRDA criteria | -confirmed 48% of probable or possible AD cases according to NINCDS-ADRDA  -confirmed 83% of AD cases where there was adequate data for validation |
| Shah et al.[12] | *THIN:  Read codes for dementia | -prevalence noted to be lower than expected, based on epidemiological surveys |

*Additional File 5 Table S4: Studies Reporting a Validation Procedure*

^‡^*ISD Scotland: Information Services Division Scotland (holds national datasets (SMR: Scottish Morbidity Records) of health outcomes/ diagnoses).*

**THIN: The Health Improvement Network; *GPRD: General Practice Research Database; *SPICE-PC: Scottish Programme for Improving Clinical Effectiveness- Primary Care*

**References**

1. Doll R, Peto R, Boreham J, Sutherland I. **Smoking and dementia in male British doctors: prospective study**. *BMJ* 2000, **320**(7242):1097-1102.

2. Guthrie B, Clark SA, McCowan C. **The burden of psychotropic drug prescribingin people with dementia: a populationdatabase study**. *Age & Ageing* 2010, **39**(5):637-642.

3. Heath CA, Mercer SW, Guthrie B. **Vascular comorbidities in younger people with dementia: a cross-sectional population-based study of 616 245 middle-aged people in Scotland**. *Journal of Neurology Neurosurgery and Psychiatry* 2015, **86**(9):959-964.

4. Imfeld P, Pernus YBB, Jick SS, Meier CR. **Epidemiology, Co-Morbidities, and Medication Use of Patients with Alzheimer's Disease or Vascular Dementia in the UK**. *Journal of Alzheimers Disease* 2013, **35**(3):565-573.

5. McGonigal G, Thomas B, McQuade C, Starr JM, MacLennan WJ, Whalley LJ. **Epidemiology of Alzheimer's presenile dementia in Scotland, 1974-88**. *BMJ* 1993, **306**(6879):680-683.

6. Newens AJ, Forster DP, Kay LDWK, Kirkup W, Bates D, Edwardson J. **Clinically diagnosed presenile dementia of the Alzheimer type in the Northern Health Region: Ascertainment, prevalence, incidence and survival**. *Psychological Medicine* 1993, **23**(3):631-644.

7. Rait G, Walters K, Bottomley C, Petersen I, Iliffe S, Nazareth I. **Survival of people with clinical diagnosis of dementia in primary care: cohort study**. *BMJ* 2010, **341**:c3584.

8. Renvoize E, Hanson M, Dale M. **Prevalence and causes of young onset dementia in an English health district**. *International Journal of Geriatric Psychiatry* 2011, **26**(1):106-107.

9. Russ TC, Gatz M, Pedersen NL, Hannah J, Wyper G, Batty GD, Deary IJ, Starr JM. **Geographical variation in dementia: Examining the role of environmental factors in Sweden and Scotland**. *Epidemiology* 2015, **26**(2):263-270.

10. Ryan DH. **Age-specific hospital incidence rates in dementia**. *Dementia* 1994, **5**(1):29-35.

11. Seshadri S, Zornberg GL, Derby LE, Myers MW, Jick H, Drachman DA. **Postmenopausal estrogen replacement therapy and the risk of Alzheimer disease**. *Archives of Neurology* 2001, **58**(3):435-440.

12. Shah SM, Carey IM, Harris T, DeWilde S, Cook DG. **The impact of dementia on influenza vaccination uptake in community and care home residents**. *Age & Ageing* 2012, **41**(1):64-69.
